# Response to Entrectinib in Differentiated Thyroid Cancer With a *ROS1* Fusion

**DOI:** 10.1200/PO.17.00105

**Published:** 2017-12-08

**Authors:** Stephen V. Liu, Laura A. Macke, Bradley S. Colton, Sikandar S. Imran, Jason Christiansen, Edna Chow-Maneval, Zachary Hornby, Pratik S. Multani

**Affiliations:** **Stephen V. Liu**, **Laura A. Macke**, **Bradley S. Colton**, and **Sikander S. Imran**, Lombardi Comprehensive Cancer Center, Georgetown University, Washington, DC; and **Jason Christiansen**, **Edna Chow-Maneval**, **Zachary Hornby**, and **Pratik S. Multani**, Ignyta, San Diego, CA.

## INTRODUCTION

The detection of gene fusions in cancer provides insight into tumorigenesis and in some cases reveals potential therapeutic targets.^1^ There are now a multitude of small-molecule inhibitors that effectively target tumors harboring specific gene fusions. With successful paradigms in place for chronic myelogenous leukemia and non–small-cell lung cancer (NSCLC), discovery of fusion events can have an immediate and significant clinical impact. Gene fusions in *ROS1*, for example, predict sensitivity to the oral tyrosine kinase inhibitor crizotinib and have led to the approval of crizotinib for the treatment of NSCLC with an *ROS1* fusion.^2^ *ROS1* fusions have also been detected in multiple other cancer types, including glioblastoma multiforme,^3^ gastric cancer,^4^ and acral lentiginous melanoma,^5^ but response to ROS1 tyrosine kinase inhibitors in tumors other than lung cancer is not well characterized. Recently, a *CCDC30*-*ROS1* fusion was identified in a case of papillary thyroid cancer^6^; this patient was successfully treated with standard therapy. There have been no other reported cases of an *ROS1* fusion in thyroid cancer. Although *ROS1* fusions have been detected in various tumor types, none were identified in 226 cases analyzed by the MSK-IMPACT (Memorial Sloan Kettering Integrated Mutation Profiling of Actionable Cancer Targets) next-generation sequencing panel^7^ or in 498 cases included in an analysis of The Cancer Genome Atlas RNA sequencing data.^8^ Here, we provide the first report to our knowledge of an *EZR*-*ROS1* fusion in a patient with metastatic papillary thyroid cancer and confirm the role of this gene fusion as a therapeutic target by describing its response to entrectinib, an orally bioavailable, CNS-active, small-molecule inhibitor of ROS1 as well as TRKA, TRKB, TRKC, and ALK.^9^

## CASE REPORT

A 50-year-old woman with no notable medical history presented with a neck mass identified as a nodular infiltrating papillary thyroid carcinoma. She underwent a total thyroidectomy, and pathologic assessment revealed a 2.2-cm focus of papillary thyroid cancer, classic variant, with negative margins, but there was penetration through the skeletal muscle and involvement of one central compartment lymph node (T3bN1aM0, TNM stage I). Postoperatively, she received adjuvant radioiodine therapy (100 mCi). Within a year, she developed mediastinal and lung recurrence. Over the next 8 years, she was treated with radioactive iodine (200 mCi after a radioactive iodine scan showed hilar uptake), stereotactic radiosurgery, and surgical resection (including a left pneumonectomy), which confirmed metastatic papillary thyroid carcinoma. In 2014, 9 years after diagnosis, she developed symptomatic brain metastases, which were treated with surgical resection and stereotactic radiation therapy. Imaging then showed metastases in the thorax, rib, liver, and brain. She began first-line systemic therapy with sorafenib, which she continued for 18 months, achieving a best response of stable disease. Her thyroglobulin levels were detectable but low and did not change notably during treatment.

With no established second-line agents for metastatic papillary thyroid cancer, the patient then participated in prescreening for the phase II basket study of entrectinib, which facilitated genomic testing of her tumor sample. As part of that testing, mRNA was isolated from a formalin-fixed paraffin-embedded sample from the resected brain metastasis and a sequencing library prepared using a custom-designed Anchored Multiplex PCR kit (ArcherDx, Boulder, CO) targeting fusions in the *NTRK1*/*2*/*3*, *ROS1*, *ALK*, and *RET* genes.^10^ Sequencing was performed on an Illumina MiSeqDx system (Illumina, San Diego, CA). This assay identified an *EZR* (exon 10)–*ROS1* (exon 34) fusion.

**Fig 1.**
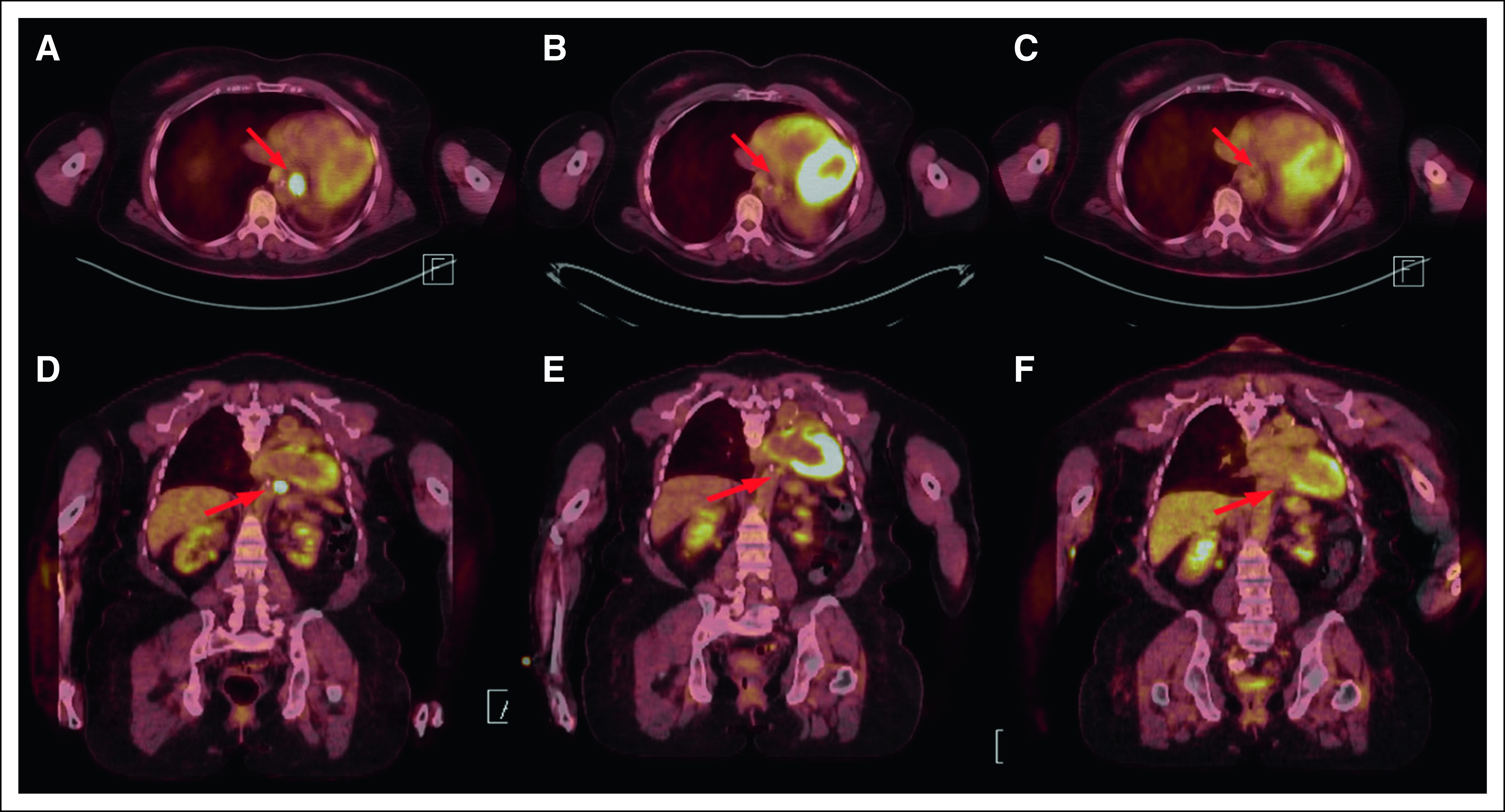
Periaortic nodule response to entrectinib. Axial and coronal images at (A, D) baseline, (B, E) 4 weeks, and (C, F) 6 months. The periaortic nodule (red arrow) was [^18^F]fluorodeoxyglucose avid at baseline (standardized uptake value, 5.23) but was not visualized at 4 weeks and remains absent.

**Fig 2.**
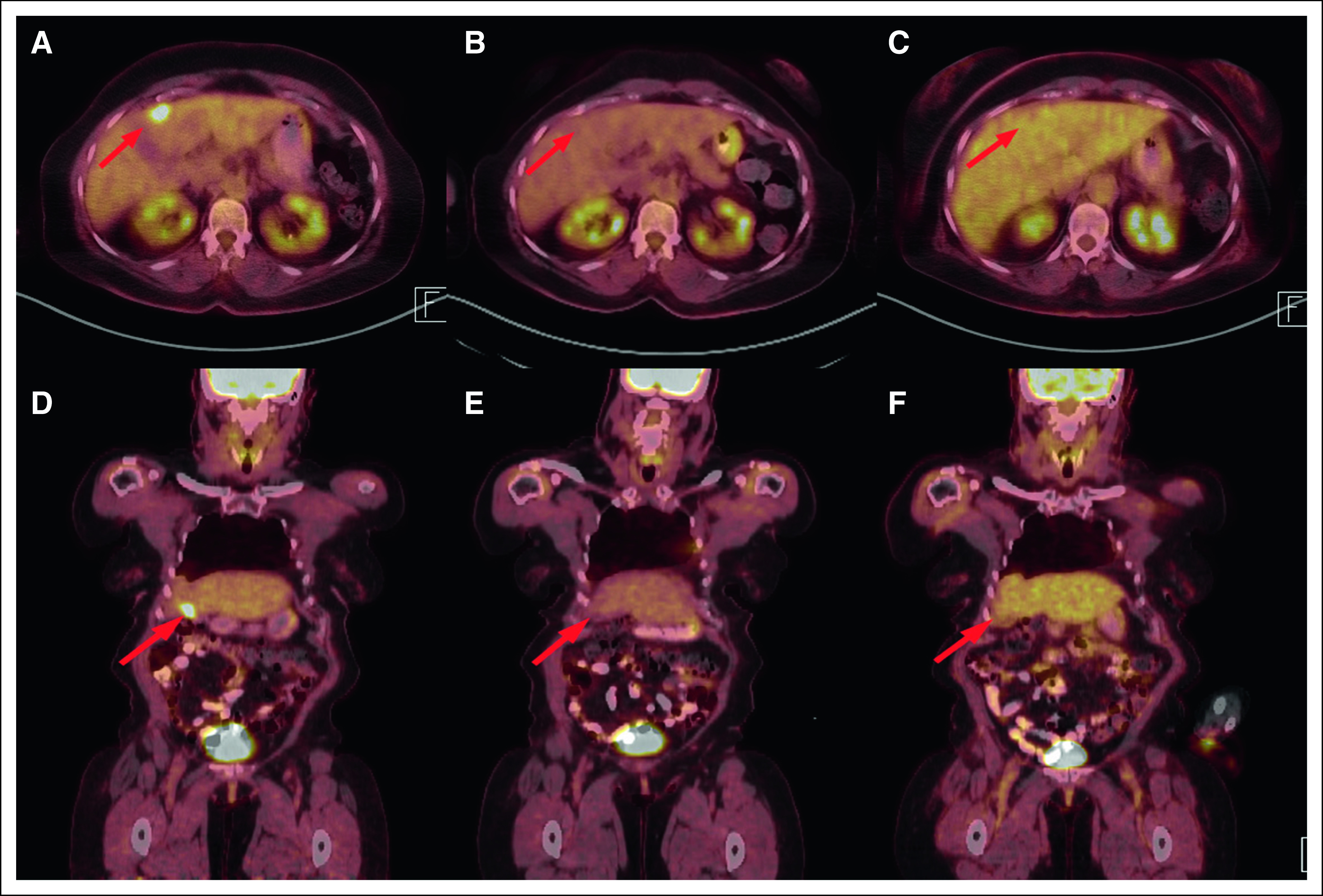
Liver metastasis response to entrectinib. Axial and coronal images at (A, D) baseline, (B, E) 4 weeks, and (C, F) 6 months. The liver metastasis (red arrow) was not well visualized on computed tomography images alone but was [^18^F]fluorodeoxyglucose avid at baseline (standardized uptake value, 12.02) but was not visualized at 4 weeks and remains absent.

The patient provided informed consent, enrolled in the phase II study of entrectinib, and started treatment in August 2016. The study was approved by the local institutional review board and is being conducted in accordance with the Declaration of Helsinki and Good Clinical Practice. At baseline, positron emission tomography/computed tomography imaging revealed a left periaortic soft tissue nodule (Figs 1A and 1D) and a solitary liver metastasis not well visualized with computed tomography alone (Figs 2A and 2D). Also identified were a left eighth rib metastasis and stable, treated brain metastases (not shown). Thyroglobulin levels did not vary during treatment. During her treatment, she developed muscle weakness and difficulty with balance, which required two dose reductions, but at the lower dose, she tolerated therapy well. After 4 weeks of therapy, the periaortic nodule was no longer visualized (Figs 1B and 1E) and remained absent 6 months after starting therapy (Figs 1C and 1F). The hypermetabolic liver metastasis had also resolved (Figs 2B and 2E) and was also absent at 6 months (Figs 2C and 2F). Her nontarget lesions in the left eighth rib and brain remained stable, overall meeting criteria for a partial response by Response Evaluation Criteria in Solid Tumors (RECIST; version 1.1).

## DISCUSSION

Detection of actionable genomic events dramatically alters the treatment plan for many cancer types. There is a growing body of evidence illustrating the activity of targeted agents across cancer types, underscoring the relevance of a genomic diagnosis.^11^ In NSCLC, the presence of a pathogenic *ROS1* rearrangement identifies candidates for treatment with crizotinib, and targeting ROS1 has been a promising strategy across fusion partners.^2,12-14^ *ROS1* gene rearrangements create fusion proteins with constitutively active kinase domains that activate downstream signaling pathways, leading to oncogenic properties in cells, including uncontrolled proliferation and resistance to cell death with prolonged tumor-cell survival. These pathways include Ras–extracellular regulated kinase for cellular proliferation and the Janus kinase–signal transducers and activators of transcription and phosphatidylinositol 3-kinase/AKT pathways, which regulate cell survival (antiapoptosis) and proliferation. *ROS1* fusion proteins may also activate the mammalian target of the rapamycin pathway, which is critical for the regulation of protein translation. Cancers that have these pathways activated tend to be more aggressive, with invasion and metastasis leading to poor patient survival.^15^ With increasing access to genomic profiling of tumors, *ROS1* rearrangements are now being identified in many other cancers. Ritterhouse et al^6^ describe a *CCDC30*-*ROS1* fusion event in papillary thyroid carcinoma, the first report in this tumor type. Here, we report another unique event: an *EZR*-*ROS1* fusion, previously described only in NSCLC.^16,17^

Although identification of these genomic aberrations across disease types is of interest, there is greater value in documenting clinical response to ROS1 inhibition. The presence of the gene fusion is clinically relevant only if it reveals a viable therapeutic target. Here, a differentiated thyroid cancer in a patient who had exhausted standard therapy was found to harbor a pathogenic fusion in *ROS1*. There are no ROS1-targeted agents approved in thyroid cancer; however, there are ongoing clinical trial opportunities, including the phase II basket trial of entrectinib (ClinicalTrials.gov identifier NCT02568267). A phase I trial of entrectinib (ClinicalTrials.gov identifier NCT02097810) has reported a favorable toxicity profile with efficacy in tumors harboring gene fusions in *NTRK*, *ROS1*, and *ALK*, including patients with CNS metastases, similar to our patient.^18^

This patient case provides additional confirmation that *ROS1* fusions are actionable targets even when observed outside of NSCLC and supports the ongoing development of small-molecule, CNS-active *ROS1* inhibitors such as entrectinib in basket study designs across multiple solid tumors. Because of the rarity of these fusion events, randomized trials may be unfeasible to conduct, but cases such as the one presented here clearly illustrate the activity of this class of targeted agents and their promise for future development through innovative clinical trial designs.
